# Vanadium-Enriched *Cordyceps sinensis,* a Contemporary Treatment Approach to Both Diabetes and Depression in Rats

**DOI:** 10.1093/ecam/neq058

**Published:** 2011-06-05

**Authors:** Jianyou Guo, ChangYu Li, Jie Wang, Yongmei Liu, Jiahui Zhang

**Affiliations:** ^1^Key Laboratory of Mental Health, Institute of Psychology, Chinese Academy of Sciences, Beijing 100101, China; ^2^Zhejiang Chinese Medical University, Hangzhou 310053, China; ^3^Molecular Biology Laboratory of Guang'anmen Hospital, China Academy of Chinese Medical Sciences, Beijing 100053, China; ^4^School of Medicine, Shandong University, Jinan 250012, China

## Abstract

This article studies a contemporary treatment approach toward both diabetes and depression management by vanadium-enriched *Cordyceps sinensis* (VECS). Streptozotocin-induced hyperglycemic rats were used in the study. After the rats were administered with VECS, a significant reduction in blood glucose levels was seen (*P* < .05) and the levels of serum insulin increased significantly (*P* < .05). At the same time, the study revealed a significant decrease in immobility with a corresponding increase in the swimming and climbing behavior in hyperglycemic rats following VECS treatment. The results described herein demonstrate that VECS is a contemporary treatment approach that advocates an aggressive stance toward both diabetes and depression management.

## 1. Introduction

Diabetes mellitus is accompanied by hormonal and neurochemical changes that can be associated with anxiety and depression [1, 2]. The prevalence of depression is ∼18% higher in diabetic patients than in the general population, and only 33% of depression cases among diabetic patients are diagnosed and treated [3, 4].These associations may be related to increased risk of depressive symptoms in individuals with diabetes, increased risk of type 2 diabetes in individuals with depressive symptoms or both. Growing evidence from clinical studies indicates that diabetic patients with major depression demonstrate poor adherence to antidiabetic regimens, have poor glycemic control and are at increased risk of retinopathy [5] and macrovascular complications [6].

The two processes of diabetes and depression negatively interact, in that depression leads to poor metabolic control whereas hyperglycemia exacerbates depression. A contemporary treatment approach advocates an aggressive stance toward both diabetes and depression management to optimize global outcome. To our knowledge, however, an algorithm incorporating the management of both has not been discovered in the literature. Thus, the present study was carried out to investigate the possible role of vanadium-enriched *Cordyceps sinensis* (VECS) in preventing depression in diabetes and influencing the longer-term course of glycemic control. One novel vanadium complex of VECS is designed and evaluated in this article.

As a potential therapeutic agent, vanadium has been attracting increasing attention. Vanadium compounds have the ability to imitate the action of insulin [7], and oral administration of inorganic vanadium salts has shown anti-diabetic activity *in vitro* [8], *in vivo* [9] and even in patients [10]. However, the toxicity associated with vanadium limits its role as a therapeutic agent for diabetic treatment [11]. Typical clinical manifestations are diarrhea, vomiting, abdominal cramps, green tongue, bronchospasm and irreversible renal excretion damage [12]. Using trace elements at lower doses, in combination with fungus, has been ascribed as one potent way to reduce toxicity associated with trace elements and maintain their effect [13, 14].

Mushrooms and primarily basidiomycetous fungi are popular and valuable foods that are low in calories and high in minerals, essential amino acids, vitamins and fibers [15, 16]. With no starch and low sugars, mushrooms might be considered the “delight of diabetics” [17]. Some of them produce substances with potential medical effects, and are called medicinal mushrooms [18–21].

*Cordyceps sinensis* is a fungus known as a traditional medicine in China. Many studies have shown that *C. sinensis* possesses hypoglycemic [22, 23] and vasorelaxant properties [24]. Another important property of fungus is the ability to take up and accumulate trace metals such as cadmium, lead, arsenic, copper, nickel, silver, chromium and mercury in the body or mycelium of the fungus [25–27]. The purpose of this study was to investigate the effect of fermented fungus of *C. sinensis* that is rich in vanadium on depression in diabetes and its influence on the course of glycemic control.

## 2. Methods

### 2.1. Chemicals

Streptozotocin (analytical grade) was purchased from Sigma. Sodium vanadate (SV; analytical grade) was purchased from Beijing Chemical Factory, China.

### 2.2. VECS

The seed of *C. sinensis* was purchased from the Agricultural Culture Collection of China. Firstly, the seed was grown at 28°C for 5 days on PDA slants (1000 mL 20% potato extract liquid, 20.0 g dextrose and 20.0 g agar). Mycelia of *C. sinensis* (five to six pieces) were transferred from a slant into 250 mL Erlenmeyer flasks containing 100 mL liquid medium (20% potato extract liquid, 2.0% dextrose, 0.1% KH_2_PO_4_ and 0.05% MgSO_4_). The culture was incubated at 27°C on a rotary shaker at 180 rmp for 4 days.

A 96-h-old liquid culture was homogenized using a sterilized blender and then inoculated to 500 mL Erlenmeyer flasks containing 300 mL of fermented culture medium (20% potato extract liquid, 2.0% dextrose, 0.1% KH_2_PO_4_, 0.05% MgSO_4_ and 0.9% NaVO_3_). The volume of inoculum was 15 mL, which was then cultivated under the same condition. The 96-h-old fermented liquid culture constituted the VECS. An ampule was filled with 4 mL of VECS stirred by a homogenizer and then was sterilized in microwave oven for 3 min. The concentration of vanadate in VECS was 0.074 M.

### 2.3. Fermented Mushroom of C. sinensis

The fermented mushroom of *C. sinensis* (FMCS) was produced using the same method to produce VECS except that there was no NaVO_3_ in the fermented culture medium.

### 2.4. SV Solution

SV (0.9 g) was dissolved in 100 mL of normal saline. An ampule was filled with 4 mL of SV and then was sterilized in a microwave oven for 3 min. The concentration of vanadate in SV was 0.074 M.

### 2.5. Animals

This study was performed in accordance with the Guidelines for Ethical Conduct in the Care and Use of Animals developed by the American Psychological Association (APA). Care was taken to minimize discomfort, distress and pain to the animals. Wistar rats of either sex weighing 150–200 g were housed in polypropylene cages (six animals/cage) under controlled temperature (27 ± 2°C) and in a natural light/dark cycle. They were fed with standard laboratory pellets. Food and water were provided *ad libitum*.

### 2.6. Drugs and Treatment

Healthy rats were made diabetic by intraperitoneal injection of streptozotocin (55 mg kg^−1^). Serum glucose levels were measured 7 days after the injection. A total of 24 animals showing hyperglycemia were selected as diabetic rats. They were randomly divided into four groups. From then on, the four groups of rats were treated daily (by oral gavage) with 4 mL of saline, VECS, FMCS and SV, respectively. The other six normal rats were treated daily (by oral gavage) with 4 mL of saline and used as the control group. At the end of the treatment (4 weeks later), the rats were fasted for 12 h, blood samples were collected from the tail vein and serum was separated. The blood glucose was analyzed with a Glucometer-4 (Bayer). Serum insulin level was determined with an enzyme-linked immunosorbant assay (ELISA) kit (Biosource, Europe).

Five weeks after diabetes induction, the rats were submitted to the forced swimming test [28]. Briefly, the rats were forced to swim individually in a cylinder (40 cm height, 15 cm diameter) containing fresh water (temperature 22 ± 2°C) up to a height of 30 cm for 15 min. This constituted the “pre-test” swim. Twenty-four hours later, each rat was re-exposed to the swimming condition in a similar environment in a 6-min “test session”. The total duration of climbing, swimming and immobility in the last 5 min of the 6-min test session was recorded for each animal. The animals were treated with 4 mL of saline, VECS, FMCS and SV, respectively, 30 min prior to the test session.

Climbing behavior consisted of upward directed movements of the forepaws along the side of the swim chamber. Swimming behavior was defined as movement (usually horizontal) throughout the swim chamber, and immobility was assigned when no additional activity was observed other than that required to keep the rat's head above water.

### 2.7. Statistical Analysis

The data were expressed as mean ± standard error of mean (SEM) and results were analyzed by ANOVA followed by Dunnett's *t* test. The *P*-value of <.05 was considered significant.

## 3. Results

### 3.1. Serum Glucose Levels in Rats

A significant reduction in blood glucose levels was seen when VECS (4 mL of VECS per day) was given to diabetic rats. The same doses of FMCS and SV did not significantly alter the glucose levels alone (Table 1).

### 3.2. Serum Insulin Levels in Rats

Table 2 depicts the serum insulin levels. Streptozotocin significantly reduced the insulin concentration. Treatment with SV had no effect on streptozotocin-induced reduction. However, the levels of serum insulin significantly increased after administration of VECS (16.69 ± 3.3 IU mL^−1^; *P* < .05) and FMCS (15.57 ± 2.1 IU mL^−1^; *P* < .05).

### 3.3. Modified Forced Swimming Test in Rats

**Figure 1:**
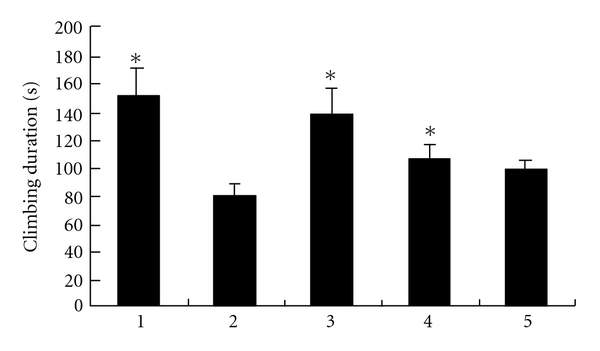
Effect of VECS on climbing time in modified forced swimming test. All bars represent mean values with vertical lines indicating SEM. Number of animals = 6. *P < .05 versus group 2. (1, Saline-treated group; 2, Steptozotocin-treated group; 3, VECS-trested group; 4, FMCS-treated group; and 5, SV-treated group).

**Figure 2:**
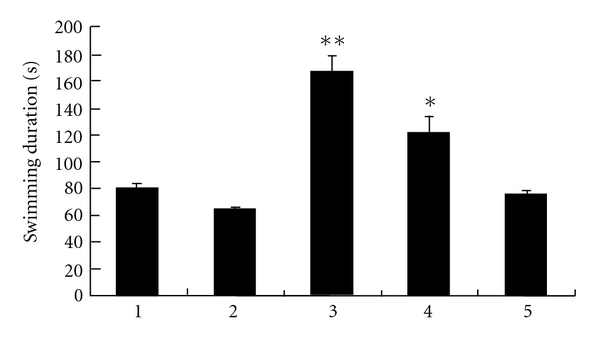
Effect of VECS on swimming time in modified forced swimming test. All bars represent mean values with vertical lines indicating SEM. Number of animals = 6. *P < .05 and **P < .01 versus group 2. (1, Saline-treated group; 2, Steptozotocin-treated group; 3, VECS-trested group; 4, FMCS-treated group; and 5, SV-treated group).

**Figure 3:**
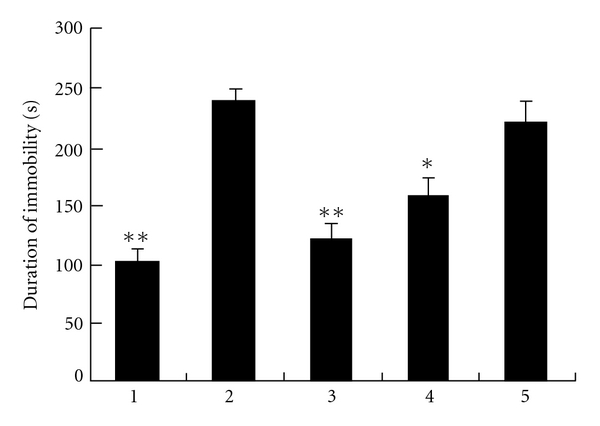
Effect of VECS on duration of immobility. All bars represent mean values with vertical lines indicating SEM. Number of animals = 6. *P < .05 and **P < .001 versus group 2. (1, Saline-treated group; 2, Steptozotocin-treated group; 3, VECS-trested group; 4, FMCS-treated group; and 5, SV-treated group).

There was a significant decrease in climbing behavior in diabetic rats. On the contrary, VECS increased climbing duration to levels similar to those of non-diabetic animals (Figure 1). No significant change in climbing behavior was observed with the treatments of FMCS and SV alone. At the same time, VECS increased swimming in diabetic rats significantly. FMCS also increased swimming. However, the same result did not occur in the SV-treated group (Figure 2). There was a subsequent reduction in immobility time with VECS treatment, whereas same doses of SV did not reduce immobility significantly.  Figure 3 shows that VECS and FMCS significantly decreased the immobility of diabetic rats in comparison with diabetic rats and the SV-treated group. However, only VECS decreased immobility to the same level seen in non-diabetic rats (*P* < .01).

## 4. Discussion and Conclusion

The presence of clinical depression and elevated depressive symptoms are higher among persons with diabetes compared with the general population. These associations may be related to increased risk of depressive symptoms in individuals with diabetes, increased risk of type 2 diabetes in individuals with depressive symptoms, or both [29, 30]. The mechanisms involved in depression are still a matter of extensive debate. Diabetes-associated depression could be related to the changes in the quality of life imposed by the chronic illness and/or its treatment, or may be a consequence of neurochemical changes induced by the disease. Studies have shown that streptozotocin-induced diabetes unbalances *γ*-aminobutyric acid, noradrenaline, serotonin and monoamine metabolite concentration in the ventromedial hypothalamus [31].

The present study characterizes the effect of a fermented fungus of *C. sinensis* rich in vanadium in a modified forced swimming test model of depression using streptozotocin-induced diabetic rats. Due to its chemical properties, in particular its greater stability, streptozotocin is the agent of choice for reproducible induction of a diabetic metabolic state in experimental animals. Streptozotocin inhibits insulin secretion and causes a state of insulin-dependent diabetes mellitus [32]. Streptozotocin-induced diabetic rats prematurely and repeatedly present more intense immobility in the forced swimming test, demonstrating their susceptibility to behavioral alterations in this animal model [33].

This is the first study reporting the antidepressant effects of fermented *C. sinensis* rich in vanadium on a modified forced swimming test, which is accepted as an animal model of depression due to its very good face and predictive validity.

**Figure 4:**
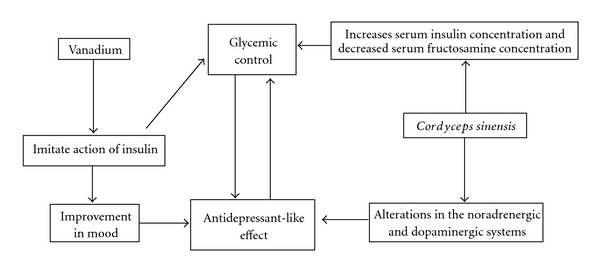
Diagram illustrating processing scheme of contemporary treatement approach of Vanadium and *Cordyceps sinensis* toward both diabetes and depression.

The hypoglycemic effect of the fermented *C. sinensis* rich in vanadium is in agreement with other similar reports [34]. It implies that the hypoglycemic effect on the hyperglycemic rats was caused by the co-effect of *C. sinensis* and vanadium (Figure 4). Vanadium is known to act as a potent insulin mimetic agent by increasing glucose transport and metabolism in skeletal muscle, liver and adipose tissue [35]. Fermented *C. sinensis* improved the diabetes-induced decrease in serum insulin concentration and attenuated the diabetes-induced increases in blood glucose concentrations [36].

Streptozotocin-induced diabetic rats prematurely and repeatedly present more intense immobility in the forced swimming test, demonstrating their susceptibility to behavioral alterations in this animal model. The results described herein demonstrate a significant decrease in immobility with a corresponding increase in the swimming and climbing behavior following VECS treatment (Figures 1, 2, and 3).

It is likely that the mechanism of the antidepressant effect of VECS is related to the co-effect of *C. sinensis* and vanadium (Figure 4). Poor glycemic control may adversely affect mood and thereby reinforce the relationship between diabetes and depression [37]. Some studies have shown the hypoglycemic functions of vanadium by insulin mimicry [38, 39]. The improved metabolic control can improve mood and that insulin mimicry may have further, favorable effects on treatment satisfaction and mood [40]. *Cordyceps sinensis* has an antidepressant-like activity and some of its constituents might act as adrenoceptor and dopamine D2 receptor agonists or noradrenaline/dopamine reuptake inhibitors [41]. Both hypoglycemic activity and antidepressant effect were caused by the co-effect of *C. sinensis* and vanadium. Neither *C. sinensis* nor vanadium could have antidepressant effect when given to the hyperglycemic rats singly.

In summary, this study has demonstrated that *C. sinensis* rich in vanadium has an antidepressant-like activity in streptozotocin-induced diabetic rats. It is a contemporary treatment approach that advocates an aggressive stance toward both diabetes and depression management.

## Figures and Tables

**Table 1:** Effect of VECS on and other treatments on serum glucose levels in streptozotocin-hyperglycemic rats.

Different groups	Serum glucose (mg dl^−1^)
Streptozotocin treated	231.9 ± 36.7^a^
Streptozotocin and SV treated	178.8 ± 10.6^a^
Streptozotocin and FMCS treated	183.2 ± 11.4^a^
Streptozotocin and VECS treated	95.8 ± 8.9^b^
Control group	100.9 ± 6.8^b^

Values are mean ± SEM. Number of animals = 6. Superscript letters in the same column indicate a statistical difference of *P* < .05.

**Table 2:** Effect of VECS on serum insulin level in streptozotocin-induced diabetic rats.

Different groups	Serum insulin (IU mL^−1^)
Streptozotocin treated	12.9 ± 1.7^a^
Streptozotocin and SV treated	11.4 ± 1.6^a^
Streptozotocin and FMCS treated	15.57 ± 2.1^b^
Streptozotocin and VECS treated	16.69 ± 3.3^b^
Saline treated	19.26 ± 2.4^b^

Values are mean ± SEM. Number of animals = 6. Superscript letters in the same column indicate a statistical difference of *P* < .05.
